# Human milk EV-miRNAs: a novel biomarker for air pollution exposure during pregnancy

**DOI:** 10.1088/2752-5309/ace075

**Published:** 2023-06-30

**Authors:** Elizabeth A Holzhausen, Allison Kupsco, Bridget N Chalifour, William B Patterson, Kelsey A Schmidt, Pari Mokhtari, Fredrick Lurmann, Andrea A Baccarelli, Michael I Goran, Tanya L Alderete

**Affiliations:** 1 Department of Integrative Physiology, University of Colorado Boulder, Boulder, CO, United States of America; 2 Department of Environmental Health Sciences, Columbia University Mailman School of Public Health, New York, NY, United States of America; 3 Department of Pediatrics, Children’s Hospital Los Angeles, Los Angeles, CA, United States of America; 4 Sonoma Technology Inc., Petaluma, CA, United States of America

**Keywords:** EV-miRNAs, breast milk, human milk, air pollution, particulate matter, near-roadway air pollution

## Abstract

Exposure to ambient and near-roadway air pollution during pregnancy has been linked with several adverse health outcomes for pregnant women and their babies. Emerging research indicates that microRNA (miRNA) expression can be altered by exposure to air pollutants in a variety of tissues. Additionally, miRNAs from breast tissue and circulating miRNAs have previously been proposed as a biomarker for breast cancer diagnosis and prognosis. Therefore, this study sought to evaluate the associations between pregnancy exposures to ambient (PM_10_, PM_2.5_, NO_2_, O_3_) and near-roadway air pollution (total NO*_x_*, freeway NO*_x_*, non-freeway NO*_x_*) with breast milk extracellular vesicle miRNA (EV-miRNA), measured at 1-month postpartum, in a cohort of 108 Latina women living in Southern California. We found that PM_10_ exposure during pregnancy was positively associated with hsa-miR-200c-3p, hsa-miR-200b-3p, and hsa-let-7c-5p, and was negatively associated with hsa-miR-378d. We also found that pregnancy PM_2.5_ exposure was positively associated with hsa-miR-200c-3p and hsa-miR-200b-3p. First and second trimester exposure to PM_10_ and PM_2.5_ was associated with several EV-miRNAs with putative messenger RNA targets related to cancer. This study provides preliminary evidence that air pollution exposure during pregnancy is associated with human milk EV-miRNA expression.

## 1. Introduction

Over 99% of the global population is exposed to levels of air pollutants in excess of the WHO guidelines [1]. In the United States, despite decreasing levels of ambient PM_2.5_ (particulate matter <2.5 *μ*m in aerodynamic diameter), significant disparities in exposure remain such that communities of color and lower socioeconomic regions experience a disproportionate burden of air pollution [2]. In particular, Latinos are exposed to higher levels of ambient and near-roadway air pollution compared to non-Hispanic whites [3, 4]. Importantly, exposure to air pollutants during sensitive windows exerts significant negative health impacts on women and children. For example, prenatal exposure to air pollutants is associated with maternal hypertension [5], gestational diabetes [6, 7], pre-term birth [8, 9], low birth weight [9, 10], and increased risk of infant adiposity [11]. Additionally, higher exposure to air pollutants has been linked with a greater risk of breast cancer among women of child-bearing age [12, 13]. The underlying mechanisms that link increased air pollution exposure with adverse health outcomes remain uncertain but may include epigenetic pathways [14, 15].

Emerging research indicates that exposure to air pollutants may impact microRNA (miRNA) expression in a variety of tissues [16–23]. miRNAs are an epigenetic mechanism that regulate post-transcriptional gene expression by degrading or repressing messenger RNAs (mRNAs). miRNAs are generated intracellularly, but can be found in many body fluids including saliva, urine, and breast milk due to active secretion via extracellular vesicles (EVs) [24]. Importantly, circulating EV-miRNAs can be transported to recipient cells and function as cell-to-cell messengers [24] that impact responses to cellular stress [25] and inflammation [26], both of which are disease mechanisms that have been associated with exposure to ambient and near-roadway air pollutants [27–29]. Indeed, there is evidence that circulating miRNAs mediate the relationships between exposure to PM_2.5_, ozone (O_3_), and nitrogen dioxide (NO_2_) with biomarkers of cardiovascular disease in adults [16, 18, 30]. Studies have also found that exposure to air pollutants including polycyclic aromatic hydrocarbons, particulate matter, O_3_, NO_2_, black carbon, and ultrafine particles are associated with changes to the expression of circulating EV-miRNA [18, 20, 21, 23] and/or circulating miRNA [19, 22].

Given the importance of the prenatal and early life periods, examining the associations between exposure to air pollutants with miRNAs may help uncover the mechanisms by which adverse environmental exposures impact maternal and child health. However, work in this area is limited. For example, one previous study examined plasma EV-miRNAs and identified 20 EV-miRNAs that were associated with exposure to air pollutants during the first trimester of pregnancy [17]. Another prior study found that human milk EV-miRNA expression was associated with maternal cigarette smoking [31] which, similar to particulate matter, is a complex mixture of inhaled pollutants. Supporting these findings, another study found that near-roadway air pollution exposure was associated with eight plasma miRNAs that are highly expressed in the breast tissue [22]. This is important since circulating miRNAs are released from organs into systemic circulation and may reflect organ-specific responses to air pollution exposure [32]. However, comparisons of miRNAs across sample type is difficult since many tissue types have varying miRNA signatures [33]. Furthermore, breast tissue and circulating miRNAs have been proposed as a biomarker for breast cancer diagnosis and prognosis [34], and miRNAs have been found to play a role in oncogenesis and metastasis [35].

While previous studies have provided support for our hypothesis that exposure to air pollutants alters human milk EV-miRNA expression, little is known about how such exposures may impact human milk EV-miRNA. For example, human milk EV-miRNAs may be a unique biomarker representing maternal exposures, and the resulting epigenetic changes have the potential to impact breast cancer risk. Further, because human milk is a primary source of early life nutrition, EV-miRNA expression in milk may have implications for infant health [36], especially as they may survive digestion in the infant gut [37–40], where they could be taken up by epithelial cells [39, 40]. Therefore, the aim of the current study was to characterize the relationships between PM_10_, PM_2.5_, NO_2_, total NO*_x_*, freeway NO*_x_*, and non-freeway NO*_x_* exposure with human milk EV-miRNA expression at 1 month postpartum in a cohort of Latina women living in Southern California. We further sought to examine whether there were sensitive windows of exposure by examining trimester-specific air pollution exposure. Finally, we explored the putative targets of EV-miRNAs that were associated with air pollution exposure to understand the biological relevance of EV-miRNA expression.

## 2. Methods

### 2.1. Study participants

The Southern California Mother’s Milk Study is an ongoing, longitudinal cohort of Latino mother-infant pairs, which recruited women from maternity clinics in Los Angeles County between 2016 and 2019. The primary aim of the Mother’s Milk Study was to analyze the impact of human milk oligosaccharides on the infant microbiome and obesity. Potential participants were eligible for inclusion in the study if they were at least 18 years of age at the time of delivery, had a healthy, singleton birth, were enrolled by 1 month postpartum, and were able to read in Spanish or English. Individuals were excluded if they were taking medications known to affect nutritional status or metabolism, reported diagnoses affecting physical or mental health, or were current tobacco or recreational drug users. The Institutional Review Boards of the University of Southern California, Children’s Hospital of Los Angeles, and the University of Colorado Boulder approved the study procedures. Written informed consent was obtained from participants prior to study enrollment.

### 2.2. Study design

Study visits occurred at 1, 6, 12, 18, and 24 months postpartum and 219 mother-infant pairs were initially enrolled in the study. Of these 219 participants, 209 mothers provided breast milk samples at the 1 month visit and 111 who had completed their 24 month visit were selected for EV-miRNA analysis. One breast milk sample was excluded from analysis because it failed sequencing, demonstrated by low (<100 000) transcriptome reads. As previously reported, those who were included in EV-miRNA analysis were slightly older and reported more breastfeedings per day when compared to those who did not undergo EV-miRNA analysis [41]. One participant, whose NO*_x_* exposure estimates were dramatically higher than average (6.4 standard deviations above the mean for total NO*_x_*), was removed from the analysis. One participant, whose visit occurred much later than the other visits (at 62 d of age) was also excluded from the analysis, resulting in a final analytical sample size of 108. Participants in this study largely belonged to a lower socioeconomic status group as determined by the modified version Hollingshead Index [42], which has been previously described [11]. Since visits occurred in Southern California, season was created as a dichotomous variable (warm/cold). Specifically, if participant visits occurred between 1 October and 31 March, they were categorized as cold season while visits that occurred between 1 April and 30 September were categorized as warm season.

### 2.3. Ambient and near roadway air pollution

Ambient air pollution (i.e., PM_10_, PM_2.5_, NO_2_, 8 hour max O_3_) was modeled using spatial interpolation of monitoring stations observations via an inverse distance-squared algorithm based on residential address. Residential address histories were assessed via a questionnaire at the 1 month visit and were geocoded using the Texas A&M Geocoder (http://geoservices.tamu.edu/Services/Geocode/). Monthly pollutant exposures were estimated using the U.S. Environmental Protection Agency’s Air Quality System (AQS, www.epa.gov/ttn/airs/airsaqs). Up to four monitoring stations within 50 km of participants’ homes were used in the spatial interpolation via an inverse distance-squared weighting (IDW2) algorithm. Near-roadway air pollution exposure (total NO*_x_*, freeway NO*_x_*, and non-freeway NO*_x_*) was estimated using dispersion modelling for roadways within 5 km of participants’ residence for all participants with a street-level geocode and sufficient local traffic volume data. Near-roadway air pollution was modelled as NO*_x_* emissions from traffic exhaust using local meteorological data, EMFAC2017 emissions factors, and the CALINE4 line dispersion model [43]. NO*_x_* was used as a surrogate for the complex mixture of particles and gases emitted by motor vehicles. This model was used to estimate the traffic impact from freeway or highway (FCC1), major collector (FCC2), minor collector (FCC3), and arterial roads (FCC4) (Streetmap Premium database, ArcGIS 10.1, Environmental Systems Research Institute Inc., Redlands, CA). Non-freeway NO*_x_* was defined as the sum of FCC2, FCC3, and FCC4. Prenatal air pollutant exposure was based on the cumulative 9 month average exposure prior to birth. Trimester-specific air pollutant exposure estimates were the exposure averages from [9,6) months prior to the birth (1st trimester), [6,3) months prior to the birth (2nd trimester), and [3,0) months prior to the birth (3rd trimester). Postnatal air pollutant exposure was based on the time between the birth and the first study visit, which occurred at approximately 1 month postpartum.

### 2.4. Breast milk collection

All breast milk samples were collected between 7 AM and 3 PM, and at least 1.5 h after a previous feeding. The time of breast milk collection was recorded. Mothers fasted for at least 1 h prior to providing the samples. Participants used an electric breast pump to provide a single, full expression from the right breast as previously described [44]. Milk was frozen at −80 °C until analysis.

### 2.5. miRNA sequencing, processing, and expression

As previously described, EVs were isolated from stored samples [31, 41]. Briefly, samples were centrifuged twice, first to remove the lipid layer and next to remove any cellular debris and apoptotic bodies. After centrifuging, the remaining volume was the skim milk volume. The ExoEasy Maxi kit (Qiagen, Germantown, MD) was used to extract EVs, and the miRNeasy Serum/Plasma Maxi kit (Qiagen, Germantown, MD) was used to isolate RNA. The RNA Clean and Concentrator-5 Kit (Zymo Research, Irvine, CA) was used to clean samples, and an Implen NanoPhotometer spectrophotometer (München, Germany) was used to measure sample purity and quality. Presence of EVs was confirmed using nanoparticle tracking analysis on the ViewSizer 3000 (Horiba Scientific), the Exo-Check Exosome Antibody Assay (System Biosciences), and transmission electron microscopy. All relevant EV characterization data have been submitted to the EV-TRACK knowledgebase (EV-TRACK ID: EV220416) [45].

The University of California San Diego performed sequencing and library preparation. Sequencing libraries were constructed using the NEBNext Small RNA Library Prep Set for Illumina (NEH, Ipswich, MA) with minor changes to the manufacturer’s protocol to optimize for low-input and cell-free RNA. Reactions were conducted at one-fifth the recommended volume, adapters were diluted 1:6, and library amplification PCR used 17 cycles. Libraries were cleaned and concentrations were quantified using the DNA Clean and Concentrator Kit (Zymo Research, Irvine, CA) and the Quant-iT PicoGreen dsDNA Assay (Invitrogen, Waltham, MA). Samples were pooled to equal volume, and size distribution was determined using a DNA HS Chip on a BioAnalyzer (Agilent Technologies, Santa Clara, CA) before size selection on a Pippin Prep instrument to remove adapter dimers and large fragments. Libraries were sequenced to approximately 1 000 000 total reads per pool using a MiSeq instrument with a Nano flow cell (Illumina Inc. San Diego, CA). Sequencing data was then used to balance the samples into new pools to conduct deeper sequencing using a HiSeq4000 instrument using single-end 75 bp runs. The proportion of ribosomal RNA (rRNA) reads was the proportion of the total input reads which were rRNA. In cell-free RNA samples, a high proportion of unmapped rRNA reads can indicate contamination by cellular material.

The ExceRpt small RNA sequencing data analysis pipeline on the Genboree Workbench (http://genboree.org/site/exrna_toolset/) [46] was used to map sequencing data using the default parameters, other than stipulating a minimum read length of 15 nucleotides and zero mismatches. Quality control was performed using the External RNA Controls Consortium guidelines [46]. One sample was removed from the analysis because it had fewer than 100 000 transcriptome reads. The trimmed mean of M method, implemented by the EdgeR package [47], was used to normalize raw EV-miRNA read counts. EV-miRNAs present in less than 70% of participants were excluded from the analysis.

### 2.6. Statistical analysis

Linear regression models were used to estimate the relationships between maternal pregnancy exposure to each air pollutant with individual natural log-transformed EV-miRNA expression (counts per million). All models were adjusted for proportion of reads mapping to rRNA from the final library, volume of skim milk, and time of milk collection as previous analyses in this cohort identified these as important factors related to sample collection and processing that were also predictive of EV-miRNA expression [41]. *A priori* hypothesized confounders, including days postpartum, mother age (years), socioeconomic status, and season of breast milk collection, were included in all models. We did not adjust for maternal pre-pregnancy BMI or BMI at 1 month postpartum since prior work in this cohort found that these characteristics were not associated with EV-miRNA expression [41]. Overall, we examined the expression of 210 EV-miRNAs and adjusted for multiple testing using the Benjamini–Hochberg (BH) procedure with a threshold of *P*_BH_ ⩽ 0.1. Next, because mammary gland development differs during pregnancy and the postnatal period [48], and there is some evidence that the effects of air pollution differ by trimester [49–51], we estimated trimester-specific associations between air pollution estimates and EV-miRNA expression, as well as the association between postnatal air pollution estimates and EV-miRNA expression.

Finally, we conducted pathway analysis to characterize the putative mRNA targets of EV-miRNAs that were associated with cumulative and trimester specific air pollutant exposure using DIANA MirPATH version 3 online software (https://dianalab.e-ce.uth.gr/html/mirpathv3/index.php?r=mirpath) [52]. Precursor miRNAs were converted to their mature counterparts for pathway analysis. We used Tarbase v7.0, a catalogue of experimentally validated miRNA-gene interactions [53], to predict mRNA targets and MirPATH to identify Kyoto Encyclopedia of Genes and Genomes (KEGG) [54] pathways with significant enrichment.

## 3. Results

### 3.1. Study population characteristics

**Figure 1.**
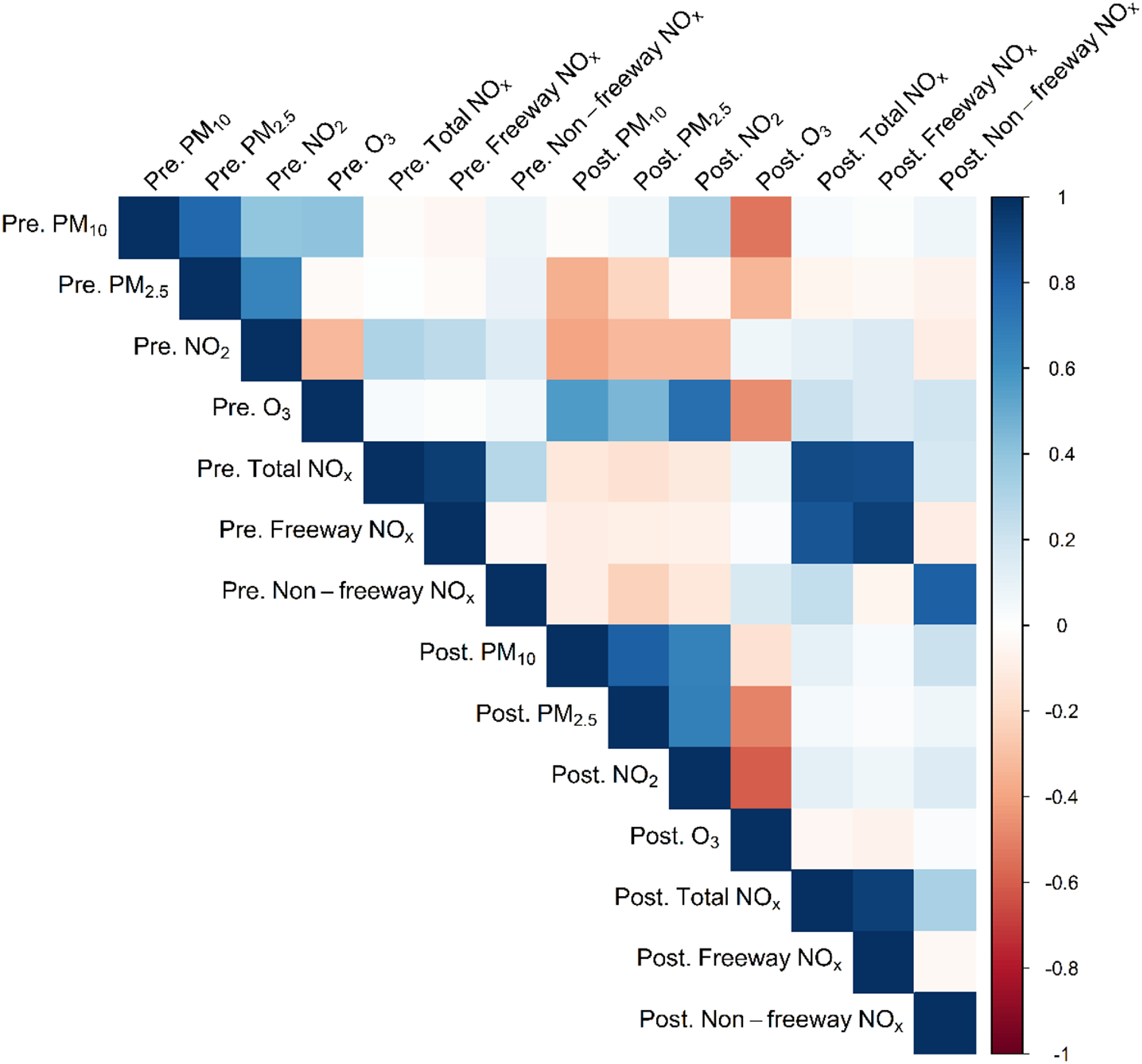
Pearson correlations between pre- and postnatal air pollution exposures.

Population characteristics are shown in table 1. Briefly, mothers were 28 years old, on average (range: 18–42 years) and had an average pre-pregnancy body mass index (BMI) of 28 kg m^−2^ with 16% having normal weight, 39% having overweight, and 45% having obesity. Additionally, 55% of infants were female and 76% were born vaginally. Visits occurred an average of 33 d postpartum and 46% of visits occurred during the warm season. Table 1 also summarizes cumulative prenatal exposure to air pollution. As expected, some air pollution measures were highly correlated, as shown in figure 1. For instance, prenatal ambient air pollutants were positively correlated with one another (e.g. the correlation between prenatal PM_2.5_ and PM_10_ was 0.78) and inversely correlated with roadway NO*_x_*. Prenatal ambient air pollutants were inversely correlated with postnatal ambient air pollutants (e.g. the correlation between pre- and postnatal NO_2_ was −0.32), while pre- and postnatal roadway NO*_x_* measures were positively correlated (e.g. the correlation between pre- and postnatal total NO*_x_* was 0.90).

**Table 1.** Characteristics of mother-infant dyads from the southern California mother’s milk study.

Maternal and infant characteristics	Mean ± SD or *N*, %
Age (years)	28.0 ± 5.6
Socioeconomic status	25.6 ± 12.2
Pre-pregnancy BMI (kg m^−2^)	28.3 ± 5.5
Infant sex (female, male, %female)	59, 49, 55%
Mode of delivery (CS, vaginal, %CS)	26, 82, 24%
Days postpartum	32.5 ± 3.4
Breastfeedings per day	6.4 ± 2.4
Season (warm, cold, %warm)	50, 58, 46%
Pregnancy ambient exposures	Mean ± SD or N, %
PM_10_ (*μ*g m^−3^)	30.0 ± 4.1
PM_2.5_ (*μ*g m^−3^)	11.9 ± 1.2
NO_2_ (ppb)	18.1 ± 2.6
O_3_ 8 hour max (ppb)	42.8 ± 3.5
Pregnancy near-roadway exposures	Mean ± SD or N, %
Total NO*_x_* (ppb)	3.9 ± 2.0
Freeway NO*_x_* (ppb)	2.1 ± 1.9
Non-freeway NO*_x_* (ppb)	1.8 ± 0.7

Baseline (1-month) characteristics of 108 Latino mother-infant dyads from the Southern California Mother’s Milk Study. Data are reported as mean and standard deviation (SD) unless otherwise noted.

Abbreviations: SD—standard deviation; BMI—body mass index; CS—cesarean section; ppb—parts per billion; *μ*g m^−3^—micrograms per cubic meter.

### 3.2. Cumulative exposure to particulate matter during pregnancy was associated with breast milk EV-miRNA expression with functional pathways related to cancer

**Figure 2.**
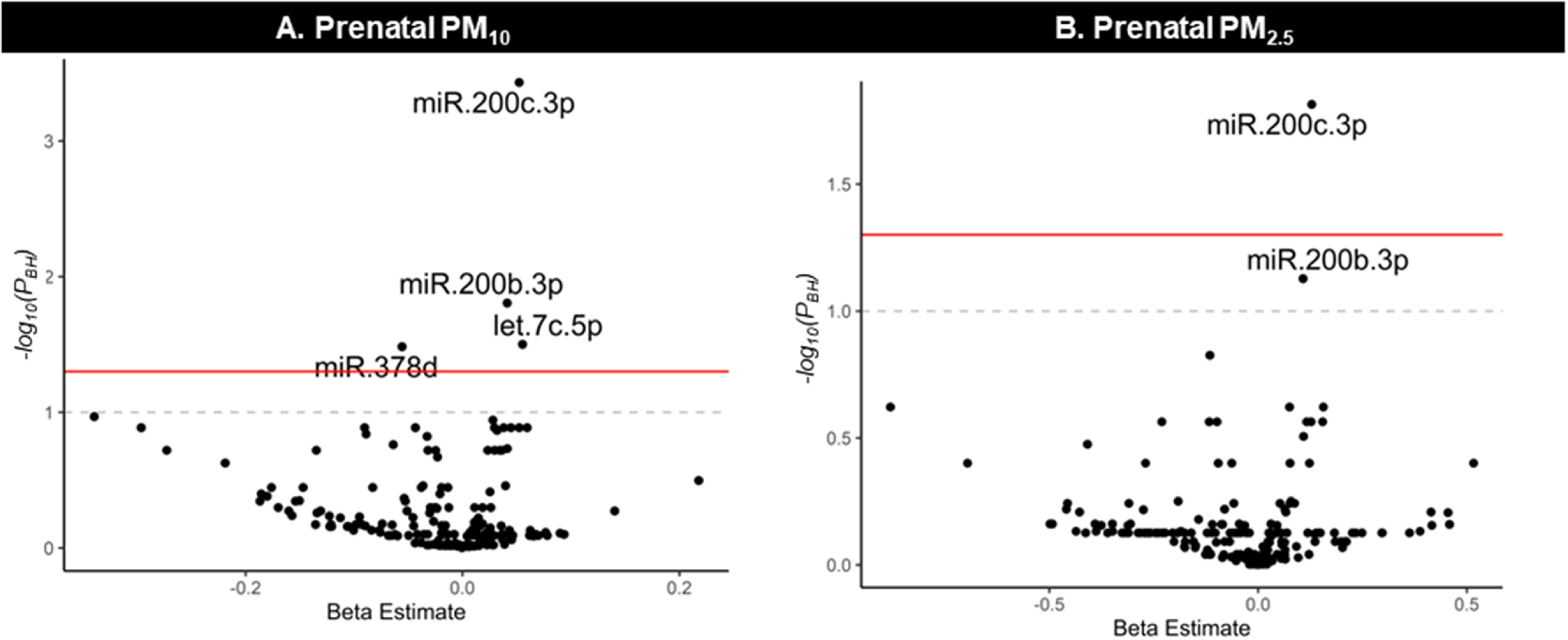
Summary of the associations between (A) prenatal PM_10_, (B) prenatal PM_2.5_ exposure with breast milk EV-miRNA expression.

**Figure 3.**
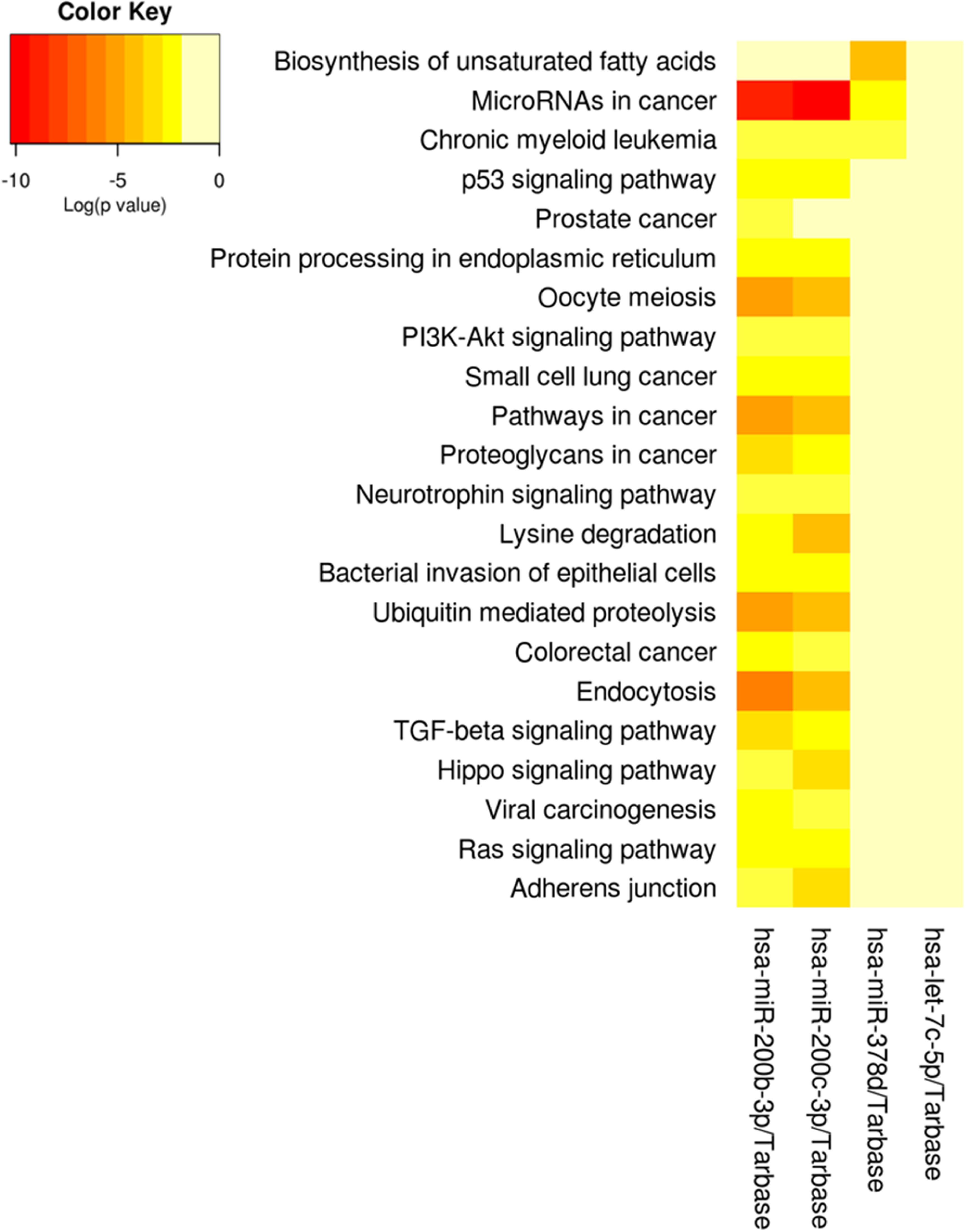
Functional annotation of breast milk EV-miRNAs that were associated with prenatal PM_10_ and prenatal PM_2.5_.

Cumulative pregnancy exposure to particulate matter was associated with breast milk EV-miRNAs after adjusting for proportion of rRNA, volume of supernatant, milk collection time, days postpartum, age, socioeconomic status, and season. Overall, pregnancy exposure to PM_10_ was positively associated with hsa-miR-200c-3p, hsa-miR-200b-3p, and let7c-5p and inversely associated with hsa-miR-378d (figure 2). Additionally, pregnancy exposure to PM_2.5_ was positively associated with hsa-miR-200c-3p and hsa-miR-200b-3p. No other statistically significant associations were observed between other cumulative pregnancy exposure to ambient air pollutants (NO_2_, O_3_) or near-roadway exposure with breast milk EV-miRNAs (supplemental table 1). We also analyzed the functional pathways of the EV-miRNAs associated with cumulative pregnancy air pollution exposure (figure 2). The following pathways were enriched among EV-miRNAs associated with both PM_10_ and PM_2.5_: miRNAs in cancer, p53 signaling pathway, and proteoglycans in cancer. However, these pathways are largely driven by the miR-200 family members, which both had more than 700 gene targets, while hsa-miR378d had 274 and let-7c-5p had none (figure 3). Given the important role that dietary intake is thought to play in the expression of breast milk EV-miRNA, we conducted a sensitivity analysis that additionally adjusted for healthy eating index (HEI) at 1-month postpartum. HEI is a composite dietary measure which assesses whether dietary intake aligns with the Dietary Guidelines for Americans [55]. Our results were largely unchanged following additional adjustment for maternal HEI (supplemental table 2). Though prior analyses in this cohort did not find a relationship between pre-pregnancy BMI or BMI at 1 month postpartum and EV-miRNA expression [41], we additionally ran sensitivity analyses adjusting for maternal pre-pregnancy BMI and found that the results were largely unchanged (supplemental table 3). As a final sensitivity analysis, all models that included NO_2_ were also adjusted for O_3_ but the results were unchanged (data not shown).

### 3.3. Trimester-specific exposure to air pollutants was associated with human milk EV-miRNA expression

**Figure 4.**
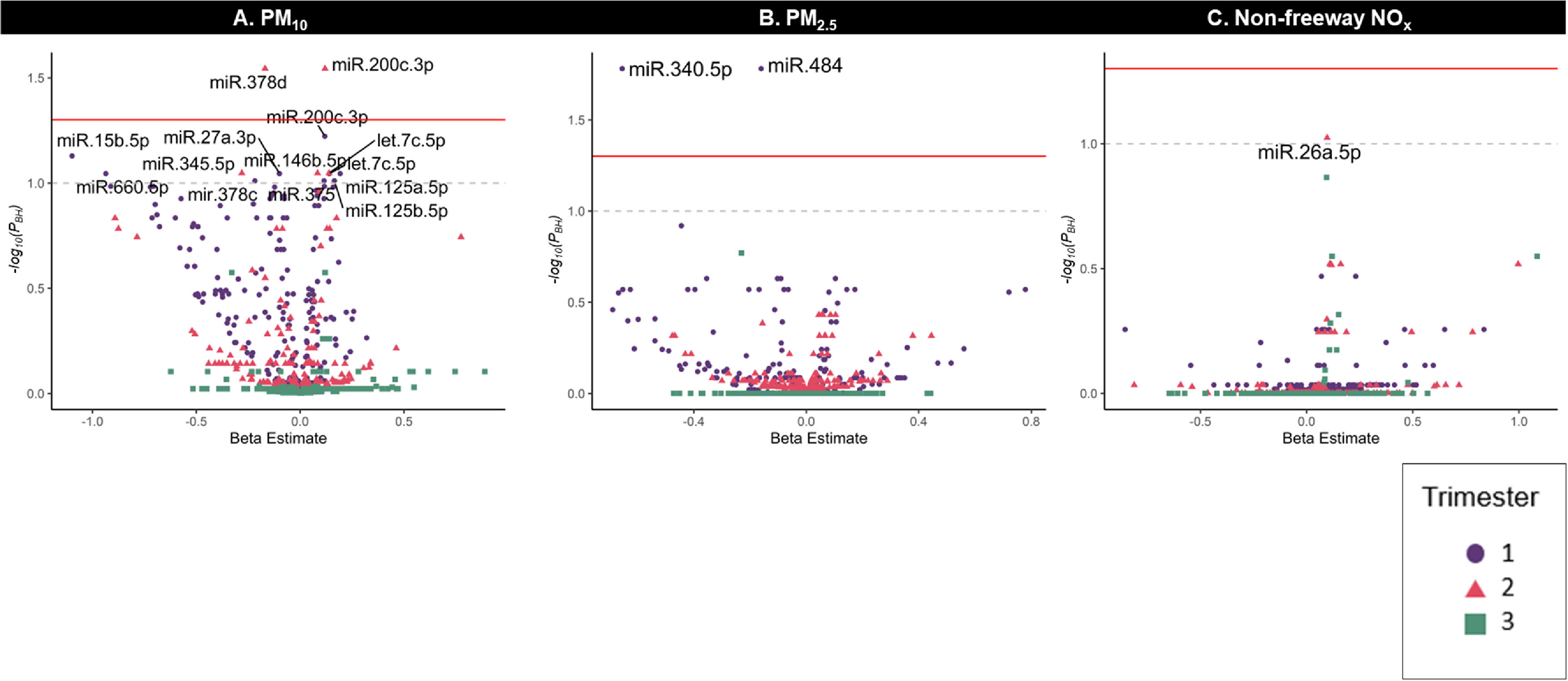
Volcano plots showing the relationship between trimester-specific (A) PM_10_, (B) PM_2.5_, and (C) non-freeway NO*_x_* exposure with the expression of several breast milk EV-miRNAs.

Overall, 15 unique EV-miRNAs were associated with at least one trimester-specific measure of air pollution exposure. PM_10_ exposure during the first trimester was associated with nine EV-miRNAs (let-7c-5p, miR-15b-5c, miR-125a-5p, miR-125b-5p, miR27a-3p, miR-200c-3p, miR-375, mir-378c, and miR-660-5p), and PM_10_ exposure in the second trimester was associated with five EV-miRNAs (let-7c-5p, miR-200c-3p, miR-345-5p, miR-378d, and miR-146b-5p) (figure 4). Additionally, first trimester PM_2.5_ exposure was inversely associated with miR-340-5p and miR-484 and second trimester non-freeway NO*_x_* was positively associated with miR-26a-5p. There were no statistically significant associations between the third trimester and PM_10_, PM_2.5_, or non-freeway NO*_x_* with EV-miRNAs. We also did not observe any statistically significant associations between trimester specific NO_2_, O_3_, total NO*_x_*, or freeway NO*_x_* with EV-miRNA expression (supplemental table 4).

We additionally analyzed the functional pathways of the EV-miRNAs that were associated with trimester specific air pollution exposure. Overall, higher exposure to PM_10_ in the first and second trimester exposure was associated with EV-miRNAs that were enriched in pathways related to cancer, including proteoglycans in cancer, miRNAs in cancer, and p53 signaling pathway (supplemental table 5). EV-miRNAs associated with higher exposure to PM_2.5_ during the first trimester were enriched in pathways including: proteoglycans in cancer, miRNAs in cancer, and p53 signaling pathway (supplemental table 6). Lastly, higher exposure to NO*_x_* in the second trimester was associated with EV-miRNAs that were enriched in pathways related to cancer and p53 signaling (supplemental table 7).

Due to structural changes in the mammary tissue following birth, we analyzed the association between postnatal air pollutant exposure and EV-miRNA expression separately. Overall, postnatal exposure to air pollutants was not associated with EV-miRNA expression (supplemental table 8).

## 4. Discussion

This study examined the relationships between pregnancy exposure to ambient and near-roadway air pollution with human milk EV-miRNA expression at 1 month postpartum. We found that higher pregnancy exposures to particulate matter, including PM_10_ and PM_2.5_, were associated with the expression of breast milk miR-200b-3p, miR-200c-3p, let-7c-5p, and miR-378d. The putative mRNA targets of these EV-miRNAs had several cancer-related functions, including p53 signaling pathway and proteoglycans in cancer. We also found that the first trimester was a sensitive period for exposure to ambient air pollutants. Specifically, human milk EV-miRNAs associated with trimester-specific exposures were enriched in functional pathways related to cancer.

In this study, increased cumulative pregnancy exposure to PM_10_ and PM_2.5_ was associated with higher levels of human milk miR-200b-3p and miR-200c-3p. These miRNAs are part of the miRNA 200 family, which is a highly conserved family with several shared biological functions including response to oxidative stress [56] and tumor metastasis [57–59]. Indeed, research has shown that expression of miRNA 200 is reduced in breast cancer tissues compared to healthy tissues [60], which may modulate chemoresistance [61, 62]. Further, overexpression of these miRNAs may inhibit the proliferation of breast cancer [60]. However, in this study, increased exposure to particulate matter was associated with increased levels of these miRNAs. Because this is a population of healthy women without a breast cancer diagnosis, these EV-miRNAs may be increasing as a compensatory mechanism, though more research is needed to assess this hypothesis. Additionally, miR-378 was associated with PM_10_ exposure during pregnancy. miR-378 has been associated with several cancers, including gastric and lung cancers [63, 64], and is thought to play a role in tumor invasion, migration, and angiogenesis [64]. Exposure to PM_10_ during the first trimester was positively associated with miR-125 expression, which is a miRNA that has been shown to have tumor suppressor functions in breast cancer [65–67]. Building on these observations, our pathway analysis found that the expression of miR-200b-3p and miR-200c-3p was associated with biological pathways related to cancer, including p53 signaling and proteoglycans in cancer. The p53 signaling pathway induces cell cycle arrest, cellular senescence, and apoptosis in response to stress signals resulting from DNA damage, oxidative stress, and activated oncogenes [54]. The proteoglycans in cancer pathways have been shown to contribute to the proliferation, adhesion, angiogenesis, and metastasis tumor processes [54, 68].

The present study also identified sensitive periods of exposure during pregnancy. As an example, exposure to PM_10_ during the first trimester was associated with nine EV-miRNAs, compared with five EV-miRNAs in the second trimester and no EV-miRNAs in the third trimester. Additionally, first trimester PM_2.5_ exposure was associated with decreased expression of miR-340-5p and miR-484, while second and third trimester PM_2.5_ was not associated with the expression of any EV-miRNAs. Extending these observations, we identified several functional pathways of EV-miRNAs that were associated with trimester specific air pollution exposure, including proteoglycans in cancer, miRNAs in cancer, and the p53 signaling pathway. Finally, we examined whether postnatal air pollution was associated with EV-miRNA expression. Our findings for postnatal PM_10_ were similar to prenatal PM_10_, although postnatal PM_10_ was also associated with decreased expression of miR-15b-5p. During the first trimester of pregnancy, estrogen, progesterone, and prolactin stimulate expansion and maturation of the mammary glands including ductal branching, alveolar morphogenesis, and secretory differentiation. In the second trimester, increasing prolactin levels trigger cellular differentiation of the mammary glands at alveolar sites. Following birth, a decrease in circulating progesterone and increasing prolactin levels stimulate secretory activation [48]. The hormonal and physiological changes inherent in each stage of mammary maturation and lactation may differ by air pollution exposure, explaining the differences in associations between human milk EV-miRNA expression with air pollution exposure across pregnancy and postpartum.

While the exact mechanisms through which pregnancy exposure to air pollutants impacts breast milk EV-miRNA expression are not yet known, current evidence suggests that miRNA expression is a response to the cellular stress and inflammation caused by exposure to ambient and near-roadway air pollutants [25–29]. In addition, ultrafine particles—a component of PM—can rapidly enter circulation via the alveoli [69]. While this study could not directly assess mechanisms by which air pollution alters expression of human milk EV-miRNA, differences in our findings between particualate matter and NO*_x_* may stem from different mechanisms of effect, which may differentially impact EV-miRNAs. Alternatively, these findings could be partly due to differences in composition of particulate matter and near-roadway air pollution (estimated using NO*_x_*), which can include metals, carbon monoxide, and ozone in addition to particulate matter. Alternatively, this may be the result of differences in exposure burden between NO*_x_* and particulate matter. We also found that EV-miRNAs that were associated with PM_2.5_ overlapped with those that were associated with PM_10_ exposure. This may be explained by the fact that PM_10_ includes exposure to both coarse and fine (PM_2.5_) particulates.

While this study identified relationships between pregnancy air pollution exposure and the expression of several human milk EV-miRNAs, there are some limitations that are worth noting. First, human milk samples were frozen between collection and analysis, which could have caused contamination by intracellular vesicles from lysed cells [70]. However, previous work on these samples showed minimal contamination by GM130, a Golgi matrix protein and marker for cellular contamination [41]. Previous studies have also shown that the methods used for EV isolation may have low EV specificity and may also include larger particles [71]. However, nanoparticle tracking analysis in a random subset of samples determined that the mean EV size was 184.5 nm and TEM visualization confirmed that the isolated EVs were of the expected size [41]. Additionally, while this study was able to characterize air pollution during pregnancy, exposures prior to pregnancy may be another important exposure period [72], which we were unable to assess. We additionally conducted a sensitivity analysis adjusting for maternal HEI, and our findings were largely unchanged. Finally, our study consisted of Latina women who largely had overweight or obesity and who intended to breastfeed for at least 6 months, which may limit the generalizability of our findings. For example, different EV-miRNAs may be identified among women with lower breastfeeding rates. However, it is especially important to understand the effects of air pollution exposure within Latino populations, as they bear a disproportionate burden of air pollution exposure and are typically understudied compared to other populations. Finally, some [12, 13] but not all [73] studies have found that increased exposure to air pollutants is linked with breast cancer risk. Notably, in the current study, we identified differences in the abundance of several miRNAs that have previously been linked to breast cancer. However, a limitation of this study is that we were unable to assess breast cancer outcomes. For this reason, future exposure studies focused on breast cancer outcomes may consider including assessment of breast milk EV-miRNAs.

## 5. Conclusions

This study provides preliminary evidence that human milk EV-miRNA expression is associated with air pollution exposure during pregnancy and that the first trimester may represent a sensitive exposure window. Several of the EV-miRNAs associated with pregnancy exposure to air pollution have previously been associated with cancer prognosis, treatment response, and metastasis. Coupled with this prior work, our results suggest that human milk EV-miRNA expression may play a role in the associations between air pollution exposure and cancer risk.

## Data Availability

The data cannot be made publicly available upon publication because they contain sensitive personal information. The data that support the findings of this study are available upon reasonable request from the authors.
